# Frequency of *Salmonella* serotypes among children in Iran*:* antimicrobial susceptibility, biofilm formation, and virulence genes

**DOI:** 10.1186/s12887-022-03614-6

**Published:** 2022-09-21

**Authors:** Akram Rezaei, Farhad B. Hashemi, Roya Rasooly Heshteli, Maryam Rahmani, Shahnaz Halimi

**Affiliations:** 1 Department of Medical Microbiology, School of Medicine, Tehran University of Medical Sciences (TUMS), 100 Poursina St., Keshavarz Blvd., Tehran, Iran; 2 Microbiology Section, Department of Pathology and Laboratory Medicine, Tehran Children Medical Center, TUMS, Tehran, Iran

**Keywords:** *Salmonella* serogroups, Serotypes, Antimicrobial susceptibility, Biofilm, Virulence genes

## Abstract

**Background/significance:**

*Salmonella* gastroenteritis causes significant morbidity among pediatric patients, mainly in developing world, such as the Middle East and North Africa (MENA) region. Concurrently, data from MENA countries like Iran, regarding prevalence of *Salmonella* serotypes, antimicrobial susceptibility, and biofilm production is scarce.

**Material & methods:**

Slide agglutination was used to determine the serogroup of 140 *Salmonella* isolates recovered from 4477 stool specimens collected from children with gastroenteritis, and isolates were serotyped by PCR assay. The antimicrobial susceptibility of isolates to five first line drugs was assessed by disk diffusion assay using CLSI guidelines. Semi-quantitative evaluation of biofilm production was done by microtiter plate assay followed by PCR detection of biofilm-associated virulence genes *csg*D, *pef*A, and *bcs*A for each isolate.

**Results:**

Nearly 94% of *Salmonella* isolates were recovered from ≤ 5-year-old patients, and 99% of isolates were non-typhoidal. While we found extensive diversity among *Salmonella* isolates, serogroup D (46%) predominated, and *Salmonella* Enteritidis (41%) was the most common serotype that showed the highest antimicrobial susceptibility rate (> 96%). For the first time in Iran, *S.* Newport serotype from human specimens was isolated. Most isolates were sensitive to all test antimicrobials, but 35% of isolates were not-typed (NT) that showed the highest resistance with 48% being resistant to ≥ 1 test antimicrobial. Majority of isolates made weak (or no) biofilm, and we found a weak association between antimicrobial susceptibility, biofilm production, or virulence genes *csg*D, *pef*A, and *bcs*A.

**Conclusions:**

The most effective measure that may control pediatric salmonellosis outbreaks is raising awareness of parents of preschoolers about food safety. Isolation of highly diverse *Salmonella* serotypes, including many commonly isolated from animals, indicates widespread contamination of the food chain. Majority of serotypes were sensitive to first-line antimicrobials, thus presently, pediatric *Salmonella* infections in this region may be controlled by conventional antimicrobials. However, despite the current trend, an imminent emergence of resistant *Salmonella* strains is foreseen, since various serotypes resistant to > 1 antimicrobial agent are typically associated with animals. Our results warrant further investigation that includes correlation analysis of clinical data regarding treatment outcomes, and serotype attributes like virulence genes.

**Supplementary Information:**

The online version contains supplementary material available at 10.1186/s12887-022-03614-6.

## Introduction

*Salmonella* species are a main cause of food-borne diseases throughout the world, particularly targeting children of pre-school age [1, 2]. A recent Global Burden of Disease study [3] has listed typhoid and paratyphoid fever as diseases that impose severe burden on resource-deprived communities [4, 5]. Moreover, several reports indicate a worrisome rising trend of non-typhoidal salmonellosis (NTS) cases in developing countries, including the Middle East and Northern Africa (MENA) [1, 5, 6]. Concurrently, data regarding prevalence of *Salmonella* serotypes, antimicrobial susceptibility, and biofilm production in countries in MENA region in Iran is scarce. A recent report that has designated *Salmonella enterica* Enteritidis (SE) as the predominant serotype in Iran, has also shown an increasing trend of antimicrobial resistance among *Salmonella* isolates recovered from patients with gastroenteritis [6].

While most cases of salmonellosis lead to a relatively mild, and self-limiting gastroenteritis, severe invasive infections warrant antimicrobial therapy and hospitalization [1, 7, 8]. Pathogenesis of *Salmonella* has been associated with several virulence genes, which have been linked to biofilm formation, tissue adhesion/invasion, antimicrobial resistance, and toxin production [5, 8, 9]. In situ growth and adhesion of *Salmonella* strains predominantly involves formation of biofilm matrix [7, 10], which are mainly composed of several polysaccharides and produced under the control of *csg*D and *pef*A genes [7]. Usually, recovery of patients treated for salmonellosis caused by biofilm-producing strains is hampered, since these strains are presumably more impervious to both antimicrobial treatment, and host immune responses [8, 11, 12]. Although primarily children are vulnerable to salmonellosis, neither the prevalence of *Salmonella* serotypes among pediatric patients in Iran, nor antimicrobial susceptibility of serotypes and their virulence characteristics have been investigated.

Moreover, the few reports that have examined the sources of *Salmonella* isolates in Iran point to a worrisome increasing trend in the antimicrobial resistance rates, accompanied by the development of multidrug resistant (MDR) *Salmonella* strains isolated from animals and food [1, 12, 13, 14]. Typically, a rise in the prevalence of *Salmonella spp.* in food sources will eventually echo as an increase in human salmonellosis cases; therefore, we have examined the prevalence of *Salmonella* serogroups and serotypes among children in Iran. Furthermore, we have assessed the antimicrobial susceptibility of the isolates, production of biofilm, and the presence of virulence genes. The epidemiologic findings of this study provide some insight for public health authorities in this region of the world, who devise strategies and policies to take effective control measures to prevent *Salmonella* outbreaks. In addition, our data on first-line drug susceptibility may help clinicians in the MENA region make optimal antimicrobial therapy choices during treatment of severe pediatric salmonellosis.

## Materials and methods

### Patient specimens, and bacterial isolates

In this cross-sectional study, a total of 140 *Salmonella* isolates were recovered from 4477 fecal specimens collected between August 2018 and September 2019 and processed by the microbiology laboratory of Tehran Children's Medical Center, an affiliate of Tehran University of Medical Sciences (TUMS). All specimens were collected from patients, who were 0–15 years old, and suffered from gastroenteritis, defined as having signs/symptoms of abdominal pain, vomiting, runny stools, and/or more than three bowl movements per day**.** All methods used in this study were carried out in accordance with relevant guidelines and regulations. Informed consent was obtained from parents of all patients and the study was approved by the TUMS Ethics Committee. After initial isolation, *Salmonella enterica* subsp. *enterica* identification was confirmed at the department of Medical Microbiology at TUMS by re-culture on xylose lysine deoxycholate (XLD) agar, followed by fifteen standard biochemical tests including, lactose and mannitol fermentation, lysine decarboxylase, methyl red, motility, H_2_S production, citrate, indole, and so forth, as described previously [15].

### Serogroup, and biotype determination; PCR identification of serotypes (Serovars), and biofilm-associated genes

The serogroup of each isolate was determined by a slide agglutination assay using *Salmonella* specific monovalent antisera (*Salmonella* kit, Sifin, Germany), and pure colonies from each isolate that cultured on trypticase soy agar (TSA) plate. Molecular identification of serotypes was carried out by polymerase chain reaction (PCR) assays using DNA of each isolate, extracted by heating at 100 °C, as previously described [16]. Table 1 shows the sequence of primers designed specifically for each serotype (serovar), and the expected amplicon sizes. The primers included *sdf* (*Salmonella enterica* Enteritidis, SEE), *stm (Salmonella enterica* Typhimurium, SET*)*, *spa (Salmonella enterica* Paratyphi B, SpTB*), fl*_*j*_B *Salmonella enterica* Infantis (SEI; Accession No. CP052776.1; Fww56-017,040), *newp*2 (*Salmonella enterica* Newport SEN), *cpcs*C (*Salmonella enterica* Paratyphi C, SEpC), and *sta*A (*Salmonella enterica* Typhi, *S.* Typhi).

**Table 1:** List of primers used to generate multifunctional PCR to identify *Salmonella* species and identify biofilm-related genes

Bacteria and Target name	Primer Sequence (5→3)	Size (bp)	Reference
*S*. Enteritidis *sdf*	F: TGTGTTTTATCTGATGCAAGAGG R: CGTTCTTCTGGTACTTACGATGAC	333	[17]
*S*. Typhimurium *stm*	F: GGAATCAATGCCCGCCAATG R: CGTGCTTGAATACCGCCTGTC	523	[18]
*S*. Infantis *fl*_*j*_*B*	F: TTGCTTCAGCAGATGCTAAG R: CCACCTGCGCCAACGCT	413	This study*
*S.* Typhi *staA*	F: TGGTTACATGACCGGTAGTC R: TAGCTGCCGCAATGGTTATG	537	[19]
*S*. ParatyphiB *Spa*	F: ACATAATGCTTTTCGTGCTCCTC R: GGCATAAATATCTTTCTCCCCTCC	384	[19]
*S.* ParatyphiC \*S*.Cholereasuis *Cspcsc*	F: TCGAGGGTTAAAGATGGGG R: TACCACACGCTAAGCAACC	708	[20]
*S*. Newport *newp2*	F: AATGGCTGGTAGCCTGTTCG R: AGGGAAAGCAAGGAACAGTAG	94	[21]
Biofilm gene *pefA*	F: CTGCGAAAGATGCCACAGAC R: CCAGCGGTACAGCTACTGAT	195	This study
Biofilm gene *csgD*	F: GTGAGTAATGCGGACTCGGT R: ACGTGGTCAGCGGATTACAG	120	This study
Biofilm gene *bcsA*	F: CATACGTCGTACCAACAGCG R: GCTGGGACATCTTAGCAACG	163	This study

^*^ https://www.ncbi.nlm.nih.gov/tools/primer-blast accession No. CP052776.1. Similar to report by Ghoddusi, A. et. al. Iranian J. of Veterinary Research, 2015, 16:3, pp. 293–297

PCR amplifications were carried out using a T100 thermal Cycler (Bio-Rad Inc.), and each 25 µL sample contained 2 µL of bacterial DNA, 0.5 µL of forward and reverse primers, 12 µL of master mix solution (Ampliqon Odense M, Denmark), as well as 10 µL of distilled H_2_O. The thermocycling conditions were as follows: Initial denaturation at 95 °C for 5 min., followed by 30 cycles of 35 s denaturation at 95 °C, and annealing phase at 59 °C for 35 s, for the *sdf*, *stm*, *spa* and *flj*B primers; 57 °C for 35 s for *newp2* primer; and 56 °C for 35 s for *cpcs*C and *sta*A primers. For all primers, the extension phase was carried out at 72 °C for 35 s, and a final 5 min extension. Amplicons were analyzed on a 1% agarose gel and visualized under UV illumination (Uvitec, Cambridge, UK).

The biotype of isolates was designated by unique combination of characteristics of each isolate, including isolate’s serotype, its antimicrobial susceptibility pattern, occurrence of virulence genes, and the strength of biofilm produced by the isolate (described below).

To detect *Salmonella* biofilm-associated genes, specific PCR primers were designed and prepared to target three genes; namely, *csg*D, *pef*A, and *bcs*A that have been linked to biofilm production [22]. Table 1 demonstrates the amplicons of these target genes i.e. 163-bp for *bcs*A, 120-bp for *csg*D, and 195-bp for *pef*A. Conditions for amplification of each target gene were as follows; initial denaturation at 95 °C for 5 min; and 34 cycles of denaturation at 95 °C for 35 s, annealing at 57 °C for 35 s and primer extension at 72 °C for 35 s; and a final extension period of 72 °C for 5 min. All primers were designed by the Primer-Blast software; (Accessed 18–09-2019; https://www.ncbi.nlm.nih.gov/tools/primer-blast).

### Determination of antibiotic susceptibility

The antimicrobial susceptibility of isolates was determined by disc agar diffusion test, which was performed on Muller-Hinton agar (Merck, Germany) plates, according to the Clinical and Laboratory Standards Institute (CLSI) guidelines [23]. Susceptibility to five different antimicrobial agents, which included two first-line drugs used against *Salmonella* infections, i.e. Ampicillin (AMP), and Trimethoprim/Sulfamethoxazole (TMP/SMX or TS) were assessed; namely, AMP (30 µg); Azithromycin (AZT; 20 µg); Ceftriaxone (CFT; 30 µg); Ciprofloxacin (CIP; 5 µg), and TS (25 µg). Intermediate susceptibility results were interpreted as resistant, and *Escherichia coli* ATCC 25,922 was utilized as a quality control organism. Double-resistance patterns were defined as resistance to two antimicrobial agents simultaneously, e.g., AMP and AZT resistance. Multidrug resistant (MDR) strains were identified as isolates that were resistant to a minimum of three antimicrobial families.

### Semi-quantitative assay for strength of biofilm production

The strength of biofilm (BF) production was measured semi-quantitatively on 96-well flat-bottom microtiter plates, as previously described [24], with a few modifications. Briefly, pure isolate colonies were suspended in 500 μL of trypticase soy broth (TSB) and diluted to a density of 0.5 McFarland in TSB, then 200 μL was added to triplicate wells. After incubation at 37 °C for 24 h, culture supernatants were decanted, wells were washed three times with 250 μL of normal saline (0.90 g/L NaCl), and biofilms were fixed by addition of 200 μL of methanol. After a 15 min incubation at room temperature (RT) the methanol was decanted, wells were air-dried, and then stained with crystal violet (150μL per well), and incubated for 15 min at RT. The plates were then rinsed 3X with tap water and air-dried. Bound stain in each well was solubilized with 150 μL of 33% (v/v) glacial acetic acid, and the optical density (OD) of samples was measured (570 nm) using a Multiskan EX reader (Labsystems, Helsinki, Finland). All samples were run in triplicate, and mock wells with no inoculum (TSB alone) were used as negative control. The cut‐off OD (OD_**c**_) for positive samples was determined as three standard deviations (SD) above the mean OD of the negative control wells. The strength of BF production of isolates was assessed by the following formulas: Strong BF = OD > (4 × OD_**c**_), Moderate BF = (2 × OD_**c**_) < OD ≤ (4 × OD_**c**_), Weak BF = OD_**c**_ < OD < 2 × OD_**c**_, and no biofilm = OD ≤ OD_c._

### Statistical analysis

All statistical analyses, including Chi-square, and Fisher's exact test, were performed using SPSS v16.0 for MS Windows, and MS Excel 2016 software, and p ≤ 0.05 was considered as significant.

## Results

### Frequency of *Salmonella* serogroups and serotypes (Serovars)

To examine the association between prevalence of *Salmonella* serogroups, and the age or sex of patients, the isolates were categorized according to serogroup, patients’ age, and gender. As shown in Table 2, serogroup D (46%) isolates were most common, whereas the serogroup C and B isolates comprised 38% and 16% of samples, respectively, while no serogroup A or E isolates were detected. Approximately 94% of isolates were recovered from patients younger than five years, and 47% of these isolates were serogroup D isolates (Table 2). Moreover, among > 5 year- old patients, the frequency of serogroup B, C, and D isolates was comparable. Although nearly 64% of isolates were collected from male patients, the relative frequency of isolates of various serogroups was not markedly different between the female and male patients (Table 2).

**Table 2:** Comparison of frequency of *Salmonella* serogroups according to gender and age group

*Salmonella* serogroup	Male	Female	Total (%)	Age (years)	
				** < 1–5**	**6–15**
	N*(%)			N*(%)	
B	15 (17)	8 (16)	**23 (16)**	20 (15)	3 (33)
C	34 (38)	19 (37)	**53 (38)**	50 (38)	3 (33)
D	40 (45)	24 (47)	**64 (46)**	61 (47)	3 (33)
**Total**	**89 (64)**	**51 (36)**	**140**	**131 (94)**	**9 (6)**

^*^ *N* = Number of isolates. All numbers > 0.5 have been rounded up

Table 3 shows that two (1%) paratyphoidal isolates; namely, *S.* Paratyphi B (SpTB) were isolated, while the vast majority (99%) of isolates were non-typhoidal. The most frequent serotype was *S.* Enteritidis (SE; 41%), followed by *S.* Newport (SN; 13%), *S.* Infantis (SI; 4%), and *S.* Typhimurium (ST; 4%); while about 36% (*N* = 51) of isolates were categorized as not-typed (NT). It is worth noting that neither *S.* Typhi, nor *S.* Paratyphi C/ Cholereasuis (serogroup C) serotypes were isolated. Although, NT serovars predominated among serogroup B and serogroup C isolates, (65% and 55%, respectively), about 11% of serogroup D isolates were comprised of NT isolates (Table 3).

**Table 3:** Comparison of frequency of *Salmonella enterica* serotypes isolated from children with gastroenteritis

*Salmonella* serogroup	Serotype**	% within each serogroup (N*)	% of Total (*N* = 140)	Total* (%)
**B**	***S*. Typhimurium**	6 (26)	**4**	**23 (16)**
	***S*. Paratyphi B**	2 (9)	**1**	
	**not-typed**	15 (65)	**11**	
	***S*. Newport**	18 (34)	**13**	
	***S*. Infantis**	6 (11)	**4**	
	**not-typed**	29 (55)	**21**	
**D**	***S*. Enteritidis**	57 (89)	**41**	**64 (46)**
	**not-typed**	7 (11)	**5**	

^*^ *N* = Number of isolates. All numbers > 0.5 have been rounded up

### Antibiotic susceptibility of serogroups and serotypes

**Fig. 1:**
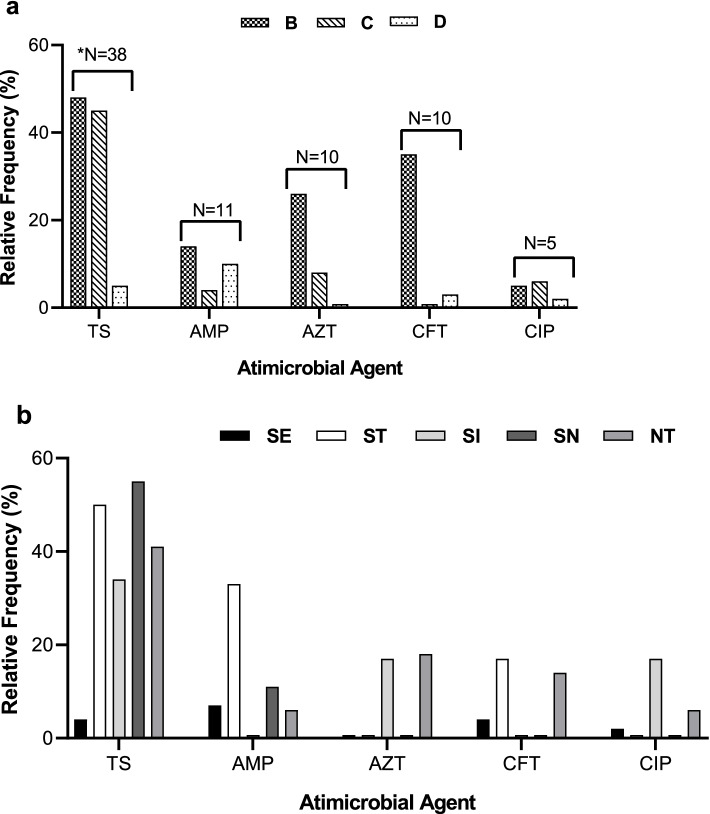
Relative frequency comparison of antimicrobial resistance rates among (**A**) serogroups, and (**B**) serotypes of *Salmonella* isolates. TS = Trimethoprim\Sulfamethoxazole, AMP = Ampicillin, AZT = Azithromycin, CFT = Ceftriaxone, CIP = Ciprofloxacin. *N = Indicates the number of isolates

In order to assess whether the first-line antibiotics are effective against *Salmonella* infections among children, we compared the antimicrobial susceptibility of isolates according to serogroup, and serotype (Fig. 1). As shown in Fig. 1a, the highest and lowest overall resistance (27%) was to trimethoprim-sulfamethoxazole (TMP-SMX), and ciprofloxacin (CIP; 4%), respectively.

Interestingly, 88% of the main serogroup D isolates were susceptible to all test antimicrobials suggesting that they were the most sensitive to antibiotics. Depending on the test antimicrobial, the susceptibility rates for serogroup D isolates ranged 91%-100%, while they showed the lowest resistance (5%) to TMP/SMX. In contrast, about 54% of serogroup B isolates (13/23), which showed the highest frequency of resistant isolates, were resistant to at least one antibiotic (Fig. 3). Furthermore, TMP/SMX resistance among serogroup C and B isolates was 45% (*N* = 24), and 48% (*N* = 11), respectively; however, 65–100% of these isolates were susceptible to AMP, AZT, CIP or CFT antimicrobials (Fig. 1a). As shown in Fig. 3, about 81% of isolates were susceptible to at least four test antimicrobial agents: namely, ampicillin (AMP), azithromycin (AZT), ceftriaxone (CFT), and ciprofloxacin (CIP). Notably, all serogroup C isolates were sensitive to ceftriaxone, while 95–98% of serogroup B and D isolates were sensitive to ciprofloxacin (Fig. 1a). Overall, isolates were least resistant to AMP, AZT and CIP with similar rates of susceptibility (approximately 93%) to these antimicrobial agents (Fig. 3).

Notably, Fig. 1b shows that *S.* Enteritidis (predominant serotype) isolates had the lowest resistance rates to various test antimicrobials (0%- 8%). In contrast, NT isolates were the least susceptible, and about 49% (*N* = 25) of them were resistant to at least one test antimicrobial (Fig. 3). Even though *S.* Newport isolates exhibited the highest overall rate of TMP/SMX resistance (44%), all the resistant isolates were susceptible to AZT, CFT, and CIP (Figs. 1b and 3). Moreover, while 66% (*N* = 4) of *S.* Typhimurium isolates were resistant to either TMP/SMX, or AMP; only one isolate was resistant to both these antimicrobials. Moreover, Fig. 3 reveals that nearly two-third (*N* = 92) of all isolates were not resistant to any of the test antimicrobials, while 21% (*N* = 29) were resistant to a single test antimicrobial. Multi-drug resistant (MDR) isolates comprised approximately 4% (*N* = 6) of isolates and were all NT isolates in serogroup B (four B-NT03, and one B-NT01 isolate), and a single serogroup C isolate (C-NT06). The more common B-NT03 MDR isolates showed resistance to antimicrobials TMP/SMX, AZT, and CFT, whereas B-NT01 isolate was also resistant to AMP (Fig. 3).

Nearly 66% (*N* = 92) of isolates were susceptible to all test antimicrobials, while resistance rate to one or > 1 test antimicrobial was 21% (*N* = 29) and 14% (*N* = 19), respectively (Fig. 3). Almost 9% (*N* = 13) of isolates were resistant to two antimicrobials (called “double resistance”), most frequently presented as “TMP/SMX & AMP” resistance (*N* = 4). While most double-resistant isolates were resistant to TMP/SMX (*N* = 10) in combination with either CFT (*N* = 3), or CIP (*N* = 2); only one isolate presented resistance to either “TMP/SMX & AZT”, “CFT & AZT”, “CFT & AMP”, or “CIP & AMP”(Additional file 1; Table S2).

### Biofilm production by *Salmonella* serogroups, and serotypes

**Fig. 2:**
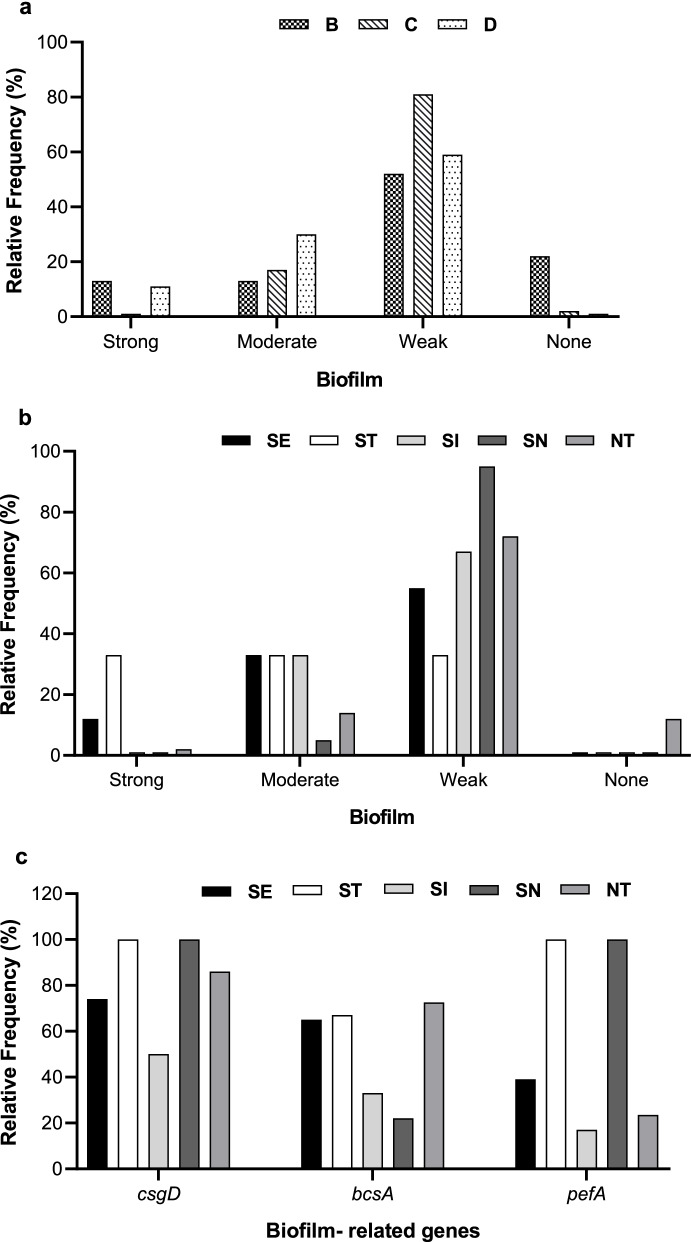
Comparison of biofilm production by *Salmonella* isolates according to (**A**) serogroups, and (**B**) serotypes; (**C**) Relative frequency of occurrence of virulence genes among various *Salmonella* serotypes. SE = *Salmonella* Enteritidis, ST = *Salmonella* Typhimurium, SI = *Salmonella* Infantis, SN = *Salmonella* Newport, NT = Not typed

**Fig. 3:**
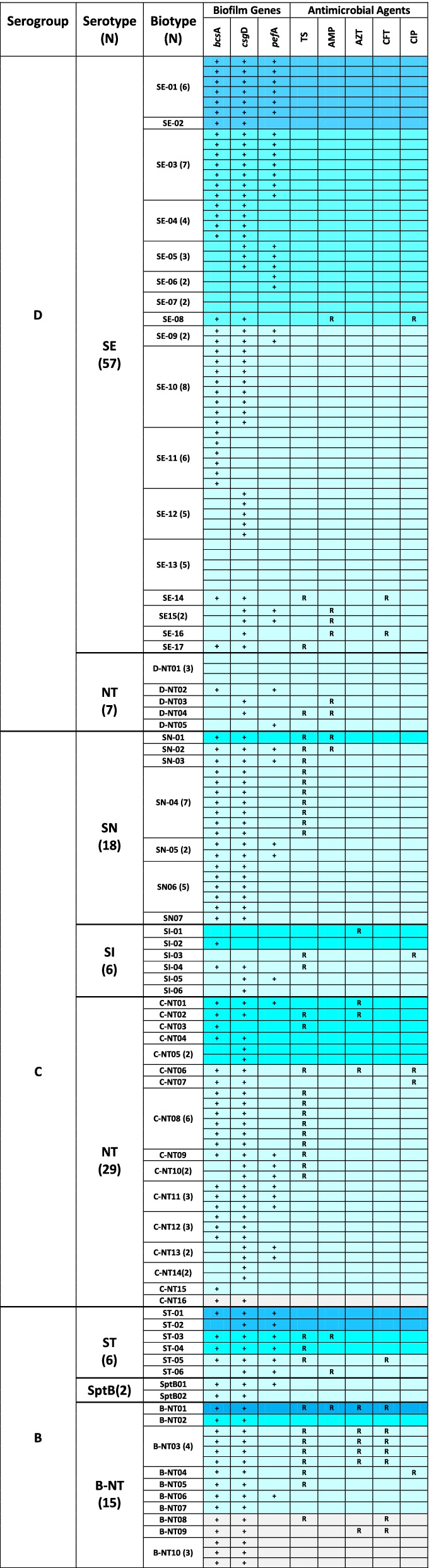
Comprehensive comparison of characteristics of *Salmonella* isolates according to serogroup, serotype, and biotype, including antimicrobial susceptibility and biofilm production. SE = *Salmonella* Enteritidis, ST = *Salmonella* Typhimurium, SI = *Salmonella* Infantis, SN = *Salmonella* Newport, NT = Not typed. All NT biotypes were categorized by designated **B**, **C**, or **D** serogroups. (R) = Resistant, ( +) = Gene detected. TS = Trimethoprim\Sulfamethoxazole, AMP = Ampicillin, AZT = Azithromycin, CFT = Ceftriaxone, CIP = Ciprofloxacin. Shades of color represent strength of biofilm production. [  = Strong biofilm formation,  = Moderate,  = Weak,  = No biofilm]

To examine possible association between virulence genes and biofilm production among *Salmonella* isolates, we compared the strength of biofilm produced by the isolates according to serogroup, as well as serotype (Fig. 2). Additionally, Fig. 3 presents a comprehensive data summarizing the biofilm production, virulence genes, and antimicrobial susceptibility profile of each isolate. As shown in Fig. 3, isolates that produced weak biofilm (WB) predominated among all serogroups, and the majority of isolates formed either WB (66%, *N* = 93), or no biofilm at all (4%, *N* = 6). In contrast, strong and moderate biofilm (MB) producers comprised 7% (*N* = 10), and 22% (*N* = 31) of all isolates, respectively. Serogroup D isolates showed the highest frequency of MB producers (30%), whereas serogroup C isolates showed the highest (81%) frequency of WB producers. In contrast, serogroup B isolates comprised the highest proportion (22%) of isolates forming no biofilm, while the frequency of strong biofilm producers was 13% among serogroup B isolates, in that order (Fig. 2a). Furthermore, Fig. 3 demonstrates that while 90% (*N* = 9) of strong biofilm producers were susceptible to all test antimicrobials, a single serogroup B isolate produced strong biofilm (NT01) and was resistant to four antimicrobials.

Additionally, Fig. 2b shows that, barring ST isolates, WB formers prevailed among various *Salmonella* serotypes, as well as the NT isolates. The highest frequency of isolates that formed WB was among SN isolates (94%), followed by NT (72%), and SI (66%) isolates. While all ST isolates produced biofilm, the ratio of weak, moderate, or strong biofilm formers among ST isolates was comparable (Fig. 2b). Likewise, the relative frequency of MB producers among the SE, SI, and ST isolates was similar (about 35%). Interestingly, ST isolates showed the highest percentage of strong biofilm producers, whereas the lowest frequency of strong biofilm formers were among the NT (2%), SN (0%) and SI (0%) isolates, which largely displayed a high antimicrobial resistance pattern (Fig. 3). Furthermore, isolates with no biofilm comprised 17% of NT isolates, which most frequent among various serotypes (Fig. 2b).

### Biofilm-related virulence genes among *Salmonella* serotypes

Figure 2c compares the relative frequency of biofilm-related virulence genes among various serotypes of *Salmonella* isolates. Additionally, to examine possible association between virulence genes, biofilm formation and the patterns of antimicrobial susceptibility among isolates, Fig. 3 collectively represents these characteristics in each isolate. Even though most WB and MB forming isolates were susceptible to test antimicrobials, overall analysis revealed no significant correlation between the presence of virulence genes and biofilm production, or resistance to test antimicrobials among serogroups or serotypes (Fig. 3).

Nevertheless, as shown in Fig. 3, 70% of strong biofilm formers (*N* = 7) were serogroup D isolates, and 85% of them carried all three virulence genes. Figure 2c demonstrates that *csgD* was the most common (83%) virulence gene among isolates, such that all ST and SN isolates carried *csgD* gene, as well as 74% of SE isolates, and 50% of SI isolates. In contrast, at frequency of 33%, *pefA* was the least frequent virulence gene, followed by *bscA* (72%), whereas 12 isolates (10 in serogroup D) did not carry any of test virulence genes. Two isolates, SI-01 and SI-03 (serogroup C), did not carry virulence genes and were resistant to AZT, and “TMP-SMX + CIP”, respectively. However, almost 84% of all isolates lacked any virulence genes, while they were susceptible to all test antimicrobials (all serogroup D isolates). Further analysis revealed that almost 91% of isolates (*N* = 128) carried ≥ 1 virulence genes, whereas 51% (*N* = 72) of isolates carried two virulence genes. As shown in Fig. 3, the number of isolates that had three virulence genes (*N* = 29; 21%( was comparable to the isolates that carried a single virulence gene (*N* = 27; 20%(.

Among the isolates positive all three virulence genes, SE serotype was the most common (52%); followed by SN (14%) and ST (14%) serotypes. Interestingly, the predominantly antibiotic sensitive SE isolates comprised 61% (*N* = 19) of moderate biofilm formers, about 37% of which carried all three virulence genes, while all MDR isolates lacked *pefA* gene, but carried *bscA* and *csgD* virulence genes. Interestingly, as shown in Fig. 3, none of D-NT isolates were positive for all three virulence genes, and *pefA* was absent in the majority of serogroup C isolates, i.e., SI and SN serotypes. Furthermore, all the six isolates that produced no biofilm carried *bscA* and *csgD* genes*;* and 80% of MDR strains (B-NT isolates) produced WB and had identical susceptibility profiles. The vast majority (89%) of strong biofilm formers were susceptible to all test antimicrobials, except for one isolate (B-NT01), which was broadly resistant (sensitive only to CIP). Serogroup D isolates comprised 78% of MB formers, which did not include any MDR isolates. In fact, nearly 80% (*N* = 23) of MB formers were sensitive to all test antimicrobials, while about 32% (*N* = 10) of them carried all three virulence genes (Fig. 3).

## Discussion

*Salmonella spp.* are leading zoonotic pathogens that cause extensive outbreaks of food-borne gastroenteritis in developing countries, such as in the MENA region, especially among children [1, 2, 6]. Although some reports from MENA have examined the prevalence and antimicrobial resistance of *Salmonella* isolates from animal and food sources, epidemiologic studies from Iran that investigate the characteristics of isolates recovered from children are scarce [1, 25–27]. We have investigated the frequency of various *Salmonella* serotypes isolated among children and assessed the association between antimicrobial susceptibility and biofilm-related virulence genes among these serotypes.

Importantly, data showing that nearly all isolates were non-typhoidal serotypes, combined with not finding any *S.* Typhi, and *S.* Paratyphi C/ Cholereasuis isolates, points to “contaminated food” as the probable source of salmonellosis among patients, as opposed to human–human transmission of infection. Remarkably, most isolates were recovered from patients < 5 years old (Table 2), which supports the notion that the policies aimed at controlling preventing salmonellosis in Iran ought to be directed at ensuring food safety for pre-school children. Due to the habitual poor personal hygiene practices of preschoolers, our finding also underscores the importance of food safety awareness among preschoolers’ parents, and day-care (and kindergarten) workers, who play a crucial role reducing the risk of salmonella infection among young children.

The data showing the predominance of *S*. Enteritidis serotype and serogroup D isolates is consistent with reports that show *S*. Enteritidis as the most frequent serotype in Iran [1, 6], and Asia [25, 26]. However, *S*. Enteritidis frequency is markedly lower among our specimens than previous studies, possibly due to the differences in patient inclusion criteria used by Sanaie et al. [6], who examined serogroup D isolates exclusively. Despite serogroup D predominance, together serogroups B and C comprised > 50% of isolates (Table 2) indicating that recently diversity among *Salmonella* spp. has expanded considerably. This expansion of diversity is further evinced by a high frequency of NT serotypes, which suggests widespread spillover of animal source *Salmonella* isolates through the food chain. For instance, for the first-time in Iran a typical animal serotype, *S*. Newport [28] was isolated from human providing additional evidence for a zoonotic spillover. These findings underscore the critical responsibility of public health authorities to ensure children food safety for by carrying out thorough and continuous monitoring of the food chain. Notably, data demonstrates that the majority of serotypes, including most common *S.* Enterica, remain susceptible to TMP/SMX and other common antimicrobials used against salmonellosis; namely, AMP and AZT for children, and CIP for adults [28, 29]. This findings is in contrast with a study that reports of the emergence *S.* Enterica isolates that are resistant to TRM/SMX, AMP, and fluoroquinolones in Iran [30]. Contrary to a recent study that has shown that 44% of *S.* Enterica isolates are resistant to CIP, we find that a 2% CIP resistance rate among *S.* Enterica isolates, which are all susceptible to TRM/SMX, and CFT [1]. However, results reveal a high rate of CFT resistance, which concurs with studies that have shown resistance to extended spectrum cephalosporins occurs more often among non-typhoidal *Salmonella* isolates [30, 31].

Generally, animal food source has been the primary route of human transmission of MDR *Salmonella,* which ultimately causes epidemics of severe human salmonellosis [11, 12, 14]. Our detection of six MDR isolates is consistent with a recent study that has reported an increase in prevalence of increased resistance among *Salmonella* isolates from animal source in Iran [32]. Our data has shown that typical animal source *Salmonella* spp., such as *S*. Newport or NT serotypes, comprised most double-resistant or MDR isolates, albeit in low frequency; indicative of a worrisome trend of widespread zoonotic spillover that can potentially develop into epidemics of resistant strains in the future. Even in the cases of double-resistant isolates, resistance to the first-line drugs such as CIP, and AZT was low, and showed similar susceptibility patterns with isolates that were resistant to a single antibiotic. Likewise, despite high TMP/SMX resistance among the typical animal source *S.* Newport isolates, they remain susceptible to CIP, AZT, and CFT antimicrobials.

Importantly, the low resistance of most common serogroup D isolates to first-line antibiotics signifies that most pediatric *Salmonella* infections in Iran, and perhaps the MENA region, may still be controlled by conventional antimicrobials. Therefore, our results suggests that at the present, children with severe salmonellosis may successfully be treated with common first-line antimicrobials. But it must be emphasized that if zoonotic contamination of food chain by typical animal *Salmonella* isolates persists in Iran, as evinced in this study, and others [14, 30, 31], standard antimicrobial therapies against salmonellosis may ultimately be rendered inadequate.

Notably, most MB producers were among the highly susceptible serogroup D isolates, while serogroup B, comprised most of “no biofilm” producers and MDR isolates. The finding that nearly all biofilm-producing serotypes are sensitive to first-line antibiotics appears contrary to several reports that have demonstrated a link between biofilm formation and acquisition of antibiotic resistance among *Salmonella* isolates [14, 24]. Remarkably, production of weak (or no) biofilm by majority of isolates combined with data showing that no MDR isolate formed strong biofilm, supports the notion that the susceptibility breakpoint values obtained would probably correspond to the antimicrobial therapy outcome of patient cases. Nonetheless, a thorough clinical study to verify the possible correlation between in vitro susceptibility results and antimicrobial therapy outcome of severe salmonellosis is warranted. It is worth mentioning that based on the susceptibility data, it stands to reason that testing TMP/SMX resistance of *Salmonella* spp. might currently have the highest clinical relevance for resource-poor microbiology laboratories in the MENA region, which may have limited access to complete antimicrobial susceptibility tests.

Interestingly, most *Salmonella* serotypes that produced strong, or medium biofilm carried all three virulence genes, but were susceptible to most test antimicrobials. Conversely, several TMP/SMX-resistant isolates that produced strong biofilm carried only two of the virulence genes. The weak association between the presence of *csg*D, *pef*A, and *bcs*A genes and biofilm formation points to the complexities of in vitro biofilm assay interpretation for *Salmonella* isolates, which warrants a thorough investigation including an in vivo model that monitors a wider array of virulence genes and their possible correlation with biofilm production. We also acknowledge a limitation of our study addressable by a correlative analysis of patients’ clinical data with *Salmonella* spp. traits, such as antimicrobial resistance, biofilm production, and virulence genes.

## Conclusions

We find that preschoolers have the highest frequency of infection with *Salmonella* spp. among pediatric patients. We also report an alarming expansion of diversity among *Salmonella* serotype probably due to contamination and zoonotic spillover into children food chain, evinced by the first-time isolation of *S.* Newport from humans in Iran. It is foreseen that persistent spillover of MDR animal *Salmonella* strains into food chain will lead to an imminent crisis that will present major challenges to clinicians treating children with infections caused by emerging highly resistant serotypes. Prompt preventive measures by public health authorities can thwart this imminent crisis by controlling the current widespread zoonotic contamination of the food chain in Iran. The highlights of this study may help epidemiologists devise policies towards effective control of pediatric salmonellosis in MENA countries such as Iran, as well as assist clinicians in making optimal therapeutic choices while treating patients with severe salmonella infections.

## Supplementary Information

**Additional file 1. Table S1.** Overall antimicrobial resistance rates according to *Salmonella* serogroup. **Table S2.** Frequency of antimicrobial double-resistance compared among *Salmonella *serogroups. **Table S3.** Comparison of frequency of virulence gene combination according to *Salmonella* serogroups.

## Data Availability

The dataset(s) supporting the conclusions of this article is(are) included within the article (and its additional file(s).

## Abbreviations

NTS: Non-typhoidal salmonellosis
SE: *Salmonella enterica* Enteritidis
MDR: Multidrug resistant
PCR: Polymerase Chain Reaction
SEE: *Salmonella enterica* Enteritidis
SET: *Salmonella enterica* Typhimurium
SpTB: *Salmonella enterica* Paratyphi B
SEI: *Salmonella enterica* Infantis
SEN: *Salmonella enterica* Newport
SEpC: *Salmonella enterica* Paratyphi C
AMP: Ampicillin
TMP/SMX or TS: Trimethoprim/Sulfamethoxazole
AZT: Azithromycin
CFT: Ceftriaxone
CIP: Ciprofloxacin
BF: Biofilm

## References

[1] Fardsanei F, Soltan Dallal MM, Douraghi M, Zahraei Salehi T, Mahmoodi M, Memariani H (2017). Genetic diversity and virulence genes of Salmonella enterica subspecies enterica serotype Enteritidis isolated from meats and eggs. Microb Pathog.

[2] Nair A, Rawool DB, Doijad S, Poharkar K, Mohan V, Barbuddhe SB (2015). Biofilm formation and genetic diversity of Salmonella isolates recovered from clinical, food, poultry and environmental sources. Infect Genet Evol..

[3] Stanaway JD, Parisi A, Sarkar K, Blacker BF, Reiner RC, Hay SI (2019). The global burden of non-typhoidal salmonella invasive disease: a systematic analysis for the Global Burden of Disease Study 2017. Lancet Infect Dis..

[4] Karkey A, Aryjal A, Basnyat B, Baker S (2008). Kathmandu, Nepal: still an enteric fever capital of the world. J Infect Dev Ctries.

[5] Andrews-Polymenis HL, Bäumler AJ, McCormick BA, Fang FC (2010). Taming the elephant: Salmonella biology, pathogenesis, and prevention. Infect Immun.

[6] Fardsanei F, Soltan Dallal MM, Douraghi M, Memariani H, Bakhshi B, Zahraei Salehi T (2018). Antimicrobial resistance, virulence genes and genetic relatedness of Salmonella enterica serotype Enteritidis isolates recovered from human gastroenteritis in Tehran Iran. J Glob Antimicrob Resist.

[7] Jajere SM (2019). A review of Salmonella enterica with particular focus on the pathogenicity and virulence factors, host specificity and adaptation and antimicrobial resistance including multidrug resistance. Vet World..

[8] Wang H, Ye K, Wei X, Cao J, Xu X, Zhou G (2013). Occurrence, antimicrobial resistance and biofilm formation of Salmonella isolates from a chicken slaughter plant in China. Food Control.

[9] Hur J, Kim JH, Park JH, Lee YJ, Lee JH (2011). Molecular and virulence characteristics of multi-drug resistant Salmonella Enteritidis strains isolated from poultry. Vet J.

[10] Crawford RW, Gibson DL, Kay WW, Gunn JS (2008). Identification of a bile-induced exopolysaccharide required for salmonella biofilm formation on gallstone surfaces. Infect Immun.

[11] Chuah LO, Shamila Syuhada AK, Mohamad Suhaimi I, Farah Hanim T, Rusul G (2018). Genetic relatedness, antimicrobial resistance and biofilm formation of Salmonella isolated from naturally contaminated poultry and their processing environment in northern Malaysia. Food Res Int.

[12] Khaltabadi Farahani R, Ehsani P, Ebrahimi-Rad M, Khaledi A. Molecular Detection, Virulence Genes, Biofilm Formation, and Antibiotic Resistance of Salmonella enterica Serotype enteritidis Isolated from Poultry and Clinical Samples. Jundishapur J Microbiol. 2018;11(10):e69504. 10.5812/jjm.69504.

[13] Vaez H, Ghanbari F, Sahebkar A, Khademi F (2020). Antibiotic resistance profiles of Salmonella serotypes isolated from animals in Iran: A meta-analysis. Iran J Vet Res..

[14] Karimiazar F, Soltanpour MS, Aminzare M, Hassanzadazar H (2019). Prevalence, genotyping, serotyping, and antibiotic resistance of isolated Salmonella strains from industrial and local eggs in Iran. J Food Safety.

[15] Mahon C, Lehman D, Manuselis G. Textbook of diagnostic microbiology-E-Book. In 2018. Chapter 19. pp. 433–4. https://books.google.com/books.

[16] Dashti AA, Jadaon MM, Abdulsamad AM, Dashti HM (2009). Heat treatment of bacteria: a simple method of DNA extraction for molecular techniques. Kuwait Med J.

[17] Trafny EA, Kozłowska K, Szpakowska M (2006). A novel multiplex PCR assay for the detection of Salmonella enterica serovar Enteritidis in human faeces. Lett Appl Microbiol..

[18] Zhai L, Yu Q, Bie X, Lu Z, Lv F, Zhang C (2014). Development of a PCR test system for specific detection of Salmonella Paratyphi B in foods. FEMS Microbiol Lett.

[19] Pratap CB (2014). Mix-infection of S. Typhi and ParaTyphi A in typhoid fever and chronic typhoid carriers: a nested PCR Based study in North India. J Clin Diagn Res..

[20] Woods DF, Reen FJ, Gilroy D, Buckley J, Frye JG, Boyd EF (2008). Rapid multiplex PCR and real-time TaqMan PCR assays for detection of Salmonella enterica and the highly virulent serovars Choleraesuis and Paratyphi C. J Clin Microbiol.

[21] Bugarel M, Tudor A, Loneragan GH, Nightingale KK (2017). Molecular detection assay of five Salmonella serotypes of public interest: Typhimurium Enteritidis, Newport, Heidelberg, and Hadar. J Microbiol Methods.

[22] Steenackers H, Hermans K, Vanderleyden J, Keersmaecker De SCJ (2012). Salmonella biofilms: An overview on occurrence, structure, regulation and eradication. Food Res Int.

[23] Hombach M, Bloemberg GV, Bottger EC (2012). Effects of clinical breakpoint changes in CLSI guidelines 2010/2011 and EUCAST guidelines 2011 on antibiotic susceptibility test reporting of Gram-negative bacilli. J Antimicrob Chemother..

[24] Zhou X, Li M, Xu L, Shi C, Shi X (2019). Characterization of antibiotic resistance genes, plasmids, biofilm formation, and in vitro invasion capacity of salmonella enteritidis isolates from children with gastroenteritis. Microb Drug Resist.

[25] Hasan TB, Lafta IJ (2021). RAPD fingerprinting and genetic diversity of salmonella spp isolated from broiler and layer flocks in Karbala. Iraq. Arch Razi Inst..

[26] Bahramianfard H, Derakhshandeh A, Naziri Z, KhaltabadiFarahani R (2021). Prevalence, virulence factor and antimicrobial resistance analysis of Salmonella Enteritidis from poultry and egg samples in Iran. BMC Vet Res.

[27] Obaidat MM, Stringer AP (2019). Prevalence, molecular characterization, and antimicrobial resistance profiles of Listeria monocytogenes, Salmonella enterica, and Escherichia coli O157:H7 on dairy cattle farms in Jordan. J Dairy Sci..

[28] Kara A, Bayram N. Susceptibility of Salmonella Isolates to Commonly Used Antimicrobial Drugs at a Tertiary Care Hospital in Izmir İzmir ‘ de Bir Üçüncü Basamak Hastanede İzole Edilen Olan Duyarlılıkları. 2017;11(4):154–8. 10.5578/ced.66936.

[29] Crump JA, Medalla FM, Joyce KW, Krueger AL, Hoekstra RM, Whichard JM (2011). Antimicrobial resistance among invasive nontyphoidal salmonella enterica isolates in the United States : National Antimicrobial Resistance Monitoring System, 1996 to 2007. Antimicrob Agents Chemother..

[30] Khademi F, Vaez H, Ghanbari F, Arzanlou M, Mohammadshahi J, Sahebkar A (2020). Prevalence of fluoroquinolone-resistant Salmonella serotypes in Iran: a meta-analysis. Pathog Glob Health.

[31] Miriagou V, Tassios PT, Legakis NJ, Tzouvelekis LS (2004). Expanded-spectrum cephalosporin resistance in non-typhoid Salmonella. Int J Antimicrob Agents.

[32] Ghoddusi A, NayeriFasaei B, ZahraeiSalehi T, Akbarein H (2019). Serotype distribution and antimicrobial resistance of salmonella isolates in human, chicken, and cattle in Iran. Arch Razi Inst..

