# Supplementary material for: Frequency of Salmonella serotypes among children in Iran: antimicrobial susceptibility, biofilm formation, and virulence genes
Source: BMC Pediatr. 2022 Sep 21;22:557. doi: 10.1186/s12887-022-03614-6 (PMC9490922; doi:10.1186/s12887-022-03614-6)
Supplement: Supplementary file 1 — Additional file 1. Table S1. Overall antimicrobial resistance rates according to Salmonella serogroup. Table S2. Frequency of antimicrobial double-resistance compared among Salmonella serogroups. Table S3. Comparison of frequency of virulence gene combination according to Salmonella serogroups. [file 12887_2022_3614_MOESM1_ESM.docx]

**Supplemental Data:**

**Table S1.** Overall antimicrobial resistance rates according to *Salmonella* serogroup.

| **No** | **Antimicrobial Agent** | **Serogroup** | | | **Total**  **N (%)** |
| --- | --- | --- | --- | --- | --- |
|  |  | **B**  **N* (%)** | **C**  **N (%)** | **D**  **N (%)** |  |
| 1 | **TMP/SMX** | 11 (29) | 24 (63) | 3 (8) | **38 (27)** |
| 2 | **AMP** | 3 (27) | 2 (18) | 6 (55) | **11 (8)** |
| 3 | **AZT** | 6 (60) | 4 (40) | 0 | **10 (7)** |
| 4 | **CFT** | 8 (80) | 0 | 2 (20) | **10 (7)** |
| 5 | **CIP** | 1 (20) | 3 (60) | 1 (20) | **5 (4)** |

[Total resistant isolates N= 74]. Numbers >0.5 were rounded up. *N= Number of isolates.

**Table S2.** Frequency of antimicrobial double-resistance compared among *Salmonella* serogroups.

| **No** | **Antimicrobial Combination** | **Serogroup** | | | **Total**  **(13)** |
| --- | --- | --- | --- | --- | --- |
|  |  | **B**  **N* (%)** | **C**  **N (%)** | **D**  **N (%)** |  |
| 1 | **AMP/CFT** | 0 | 0 | 1 (100) | **1** |
| 2 | **AMP/TS** | 1 (25) | 2 (50) | 1 (25) | **4** |
| 3 | **AZT/TS** | 0 | 1 (100) | 0 | **1** |
| 4 | **AMP/CIP** | 0 | 0 | 1 (100) | **1** |
| 5 | **TS/CIP** | 1 (50) | 1 (50) | 0 | **2** |
| 6 | **TS/CFT** | 2 (66) | 0 | 1 (33) | **3** |
| 7 | **AZT/CFT** | 1 (100) | 0 | 0 | **1** |

Numbers >0.5 were rounded up. *N= Number of isolates

**Table S3.** Comparison of frequency of virulence gene combination according to *Salmonella* serogroups.

| **Total (103)** | **Serogroup** | | | **Virulence Genes** | **No** |
| --- | --- | --- | --- | --- | --- |
|  | **D**  **N (%)** | **C**  **N (%)** | **B**  **N* (%)** |  |  |
| **90** | 31 (35) | 38 (42) | 21 (23) | ***bcs* A + *csg* D** | 1 |
| **12** | 5 (42) | 5 (42) | 2 (17) | ***pef* A +*csg* D** | 2 |
| **1** | 1 (100) | 0 | 0 | ***pef* A + *bcs* A** | 3 |

Numbers >0.5 were rounded up. *N= Number of isolates.
